# Cdh1 inhibits WWP2-mediated ubiquitination of PTEN to suppress tumorigenesis in an APC-independent manner

**DOI:** 10.1038/celldisc.2015.44

**Published:** 2016-02-02

**Authors:** Jia Liu, Lixin Wan, Jing Liu, Zhu Yuan, Jinfang Zhang, Jianfeng Guo, Marcos Malumbres, Jiankang Liu, Weiguo Zou, Wenyi Wei

**Affiliations:** 1 Center for Mitochondrial Biology and Medicine, The Key Laboratory of Biomedical Information Engineering of Ministry of Education, School of Life Science and Technology and Frontier Institute of Life Science, FIST, Xi’an Jiaotong University, Xi’an, China; 2 Cardiovascular Research Center, Xi'an Jiaotong University School of Medicine, Xi'an, China; 3 Department of Pathology, Beth Israel Deaconess Medical Center, Harvard Medical School, Boston, MA, USA; 4 State Key Laboratory of Biotherapy and Cancer Center, Sichuan University/Collaborative Innovation Center of Biotherapy, West China Hospital, Sichuan University, Chengdu, China; 5 Department of Obstetrics and Gynecology, Union Hospital, Tongji Medical College, Huazhong University of Science and Technology, Wuhan, China; 6 Cell Division and Cancer group, Spanish National Cancer Research Centre (CNIO), Madrid, Spain; 7 State Key Laboratory of Cell Biology, Institute of Biochemistry and Cell Biology, Shanghai Institutes for Biological Sciences, Chinese Academy of Sciences, Shanghai, China

**Keywords:** Cdh1, WWP2, PTEN, Akt, tumorigenesis

## Abstract

Anaphase-promoting complex/cyclosome/Cdh1 is a multi-subunit ubiquitin E3 ligase that drives M to G1 cell cycle progression through primarily earmarking various substrates for ubiquitination and subsequent degradation by the 26S proteasome. Notably, emerging evidence suggested that Cdh1 could also function in various cellular processes independent of anaphase-promoting complex/cyclosome. To this end, we recently identified an anaphase-promoting complex/cyclosome-independent function of Cdh1 in modulating osteoblast differentiation through activating Smurf1, one of the NEDD4 family of HECT domain-containing E3 ligases. However, it remains largely unknown whether Cdh1 could exert its tumor suppressor role through similarly modulating the E3 ligase activities of other NEDD4 family members, most of which have characterized important roles in tumorigenesis. Here we report that in various tumor cells, Cdh1, conversely, suppresses the E3 ligase activity of WWP2, another NEDD4 family protein, in an anaphase-promoting complex/cyclosome-independent manner. As such, loss of Cdh1 activates WWP2, leading to reduced abundance of WWP2 substrates including PTEN, which subsequently activates PI3K/Akt oncogenic signaling to facilitate tumorigenesis. This study expands the non-anaphase-promoting complex/cyclosome function of Cdh1 in regulating the NEDD4 family E3 ligases, and further suggested that enhancing Cdh1 to inhibit the E3 ligase activity of WWP2 could be a promising strategy for treating human cancers.

## Introduction

The anaphase-promoting complex/cyclosome (APC/C, also named APC) is a 1.5-megadalton ubiquitin E3 ligase complex encompassing at least 14 different subunits [1, 2]. APC controls cell cycle progression mainly in the M and G1 phases through forming two distinct subcomplexes, APC^Cdh1^ and APC^Cdc20^, to promote timely ubiquitination and subsequent degradation of mitotic cyclins and other cell cycle regulators [3]. Cdh1, which associates with the APC core complex as one of the two co-activators, recruits substrates to the APC core complex largely via KEN Box or Destruction Box (D-box) motifs found in most APC^Cdh1^ substrates [4, 5]. On the other hand, Cdc20, another well-characterized APC cofactor, primarily participates in the metaphase to anaphase transition by targeting D-box-containing substrates for ubiquitination [6]. Besides its conventional roles in cell cycle regulation, Cdh1 has recently been found to also have critical roles in a wide spectrum of cellular processes including DNA damage repair [7, 8], cellular metabolism [9], cell migration[10] and neuronal development [11, 12]. More importantly, mice deficient in *Cdh1* were embryonic lethal, while *Cdh1* heterozygous mice displayed a decrease in survival and were more susceptible to developing epithelial tumors [13], suggesting a tumor suppressor role for Cdh1. This genetic evidence was further supported by recent studies revealing a decrease of Cdh1 expression in various human tumor tissues [7, 14, 15]. Moreover, besides functioning as a co-activator for the APC core complex, we recently identified a novel, APC-independent role for Cdh1 by disrupting the intermolecular interaction of Smurf1 dimers, leading to Smurf1 activation [16]. This finding expanded the functional territory of Cdh1 in osteoblast differentiation. However, it remains largely unclear whether Cdh1 could also modulate other NEDD4 family of HECT domain-containing E3 ligases and whether Cdh1 could do so in an APC-dependent or APC-independent manner.

As one of the nine NEDD4 family of E3 ligase proteins, WWP2 contains an N-terminal membrane targeting C2 domain, four internal double tryptophan (WW) domains and a C-terminal HECT domain that confers E3 ligase activity [17]. WWP2 regulates various biological processes through targeting its substrates for ubiquitination and subsequent degradation. For example, WWP2 controls PTEN stability to influence the PI3K/Akt signaling pathway in tumorigenesis [18]; modulates cellular metastasis by triggering the turnover of Smad proteins [19]; and negatively regulates innate immune and inflammatory responses via targeting TRIF for ubiquitination and destruction [20]. Recent studies have also demonstrated that like many NEDD4 family members including Smurf1, WWP2 undergoes auto-ubiquitination to accelerate its own turnover [21]. Moreover, WWP2 may also adopt an auto-inhibitory conformation [22], a regulatory mechanism shared by various NEDD4 family of E3 ligases, including Smurf2 [22] and Itch [23]. Furthermore, similar to Smurf2, the N-terminal C2 domain of WWP2 may interact with the C-terminal HECT domain within the same molecule, which forms a closed conformation to either block the access of substrates to the WW domain, or prevent E2 recruitment [17], leading to auto-suppression of its E3 ligase activity. Hence, releasing this auto-suppression could lead to activation of various members of the NEDD4 family of E3 ligases. To this end, activation of the TGF-β signaling pathway has been reported to result in Smad7 accumulation, further leading to elevated interaction of Smad7 with Smurf2 to disrupt the intramolecular inhibition of Smurf2, thereby activating the E3 ligase activity of Smurf2 [22]. However, it remains largely uncharacterized whether a similar auto-suppressive mechanism operates to govern the WWP2 E3 ligase activity and how WWP2 E3 ligase activity is regulated by upstream factors.

As a natural extension of our previous report identifying an APC-independent role of Cdh1 in disrupting the intermolecular interaction of Smurf1 dimers [16], here we demonstrate that Cdh1 also regulates WWP2 E3 ligase activity independent of the APC core complex. However, opposite to Cdh1-mediated augmentation of the enzymatic activity of Smurf1, Cdh1 suppresses WWP2 by binding to both the C2 and HECT domains of WWP2, thereby locking WWP2 in its auto-inhibitory conformation. As a result, Cdh1 modulates the PI3K/Akt signaling pathway by influencing WWP2-mediated degradation of PTEN in cancer cells to govern tumorigenesis.

## Results

### Depletion of *Cdh1* leads to the activation of WWP2 ubiquitin E3 ligase activity

**Figure 1:**
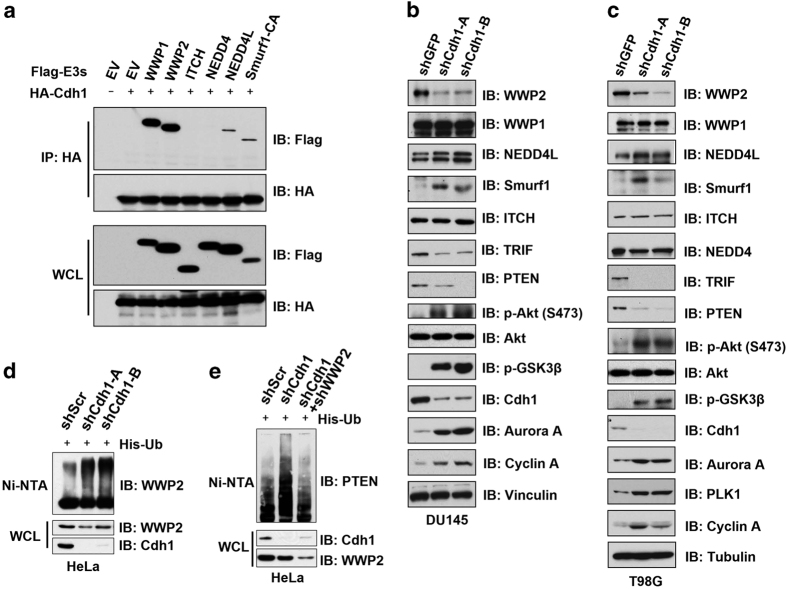
Cdh1 negatively regulates the E3 ligase function of WWP2. (**a**) Immunoblot (IB) analysis of whole-cell lysates (WCL) and immunoprecipitates (IP) derived from 293 T cells transfected with various Flag-tagged E3 ligase constructs together with HA-Cdh1. Thirty-six hours post transfection, the cells were pretreated with 10 μM MG132 for 10 h before collecting. The enzymatically inactive Smurf1/CA mutant was used to enhance Smurf1 expression to match protein abundance comparable to other NEDD4 family proteins. (**b**, **c**) IB analysis of WCL derived from DU145 (**b**) or T98G (**c**) cells infected with the indicated lentiviral shRNA constructs. The infected cells were selected with 1 μg ml^−1^ puromycin for 72 h to eliminate the noninfected cells before collecting for IB analysis. (**d**, **e**) IB analysis of WCL and subsequent Ni-NTA pull-down in 6 M guanidine-HCl-containing buffer derived from HeLa cells infected with the indicated shRNA constructs. The infected cells were selected with 1 μg ml^−1^ puromycin for 72 h to eliminate the noninfected cells before collecting. The cells were pretreated with 10 μM MG132 for 10 h before collecting. shRNA, short hairpin RNA.

We have previously demonstrated that Cdh1 could interact with Smurf1 to augment its E3 ligase activity [16]. Therefore, we further determined whether other NEDD4 family members, including WWP1, WWP2, NEDD4, NEDD4L and ITCH, could also bind Cdh1. Notably, we found that besides Smurf1 [16], WWP1, WWP2 and NEDD4L also bound to Cdh1 in cells (Figure 1a). To further investigate whether Cdh1 could also control the E3 ligase activity of these Cdh1-interacting NEDD4 family of E3 ligases, we depleted endogenous *Cdh1* in multiple cancer cell lines (Figure 1b and c and Supplementary Figure S1a and c) and examined the protein levels of NEDD4 family members interacting with Cdh1. Importantly, depletion of endogenous *Cdh1* in these cancer cell lines significantly suppressed the protein levels of WWP2 but not other NEDD4 family proteins examined (Figure 1b and c and Supplementary Figure S1a and c). As Cdh1 protein abundance fluctuates across cell cycle [3], we also examined whether WWP2 abundance changes during cell cycle progression. Notably, we found a positive correlation between Cdh1 and WWP2 protein abundance in thymidine blocked and released HeLa cell lysates (Supplementary Figure S1d), suggesting that Cdh1 could regulate WWP2 abundance in a cell cycle-dependent manner.

Conversely, consistent with our previous report [16], depletion of *Cdh1* led to an accumulation of inactive Smurf1 (Figure 1b and c) that may be largely owing to a loss of Cdh1-mediated activation of Smurf1 (Supplementary Figure S2a) [16]. This finding is consistent with previous reports that NEDD4 family ubiquitin E3 ligases undergo auto-ubiquitination to control their self-turnover [17]. In this circumstance, elevated protein abundance of NEDD4 family members such as Smurf1 may indicate decreased E3 ligase activities, whereas reduced protein abundance of NEDD4 family members might suggest augmented E3 ligase activities. Hence, the observed downregulation of WWP2 suggested that compared with control cells, WWP2 might be more active in *Cdh1*-depleted cells (Supplementary Figure S2b). However, it is also possible that depletion of *Cdh1* might lead to an upregulation of other upstream regulator(s) to indirectly reduce WWP2 abundance (Supplementary Figure S2c).

To uncover the molecular mechanism underlying the observed reduction of WWP2 abundance owing to *Cdh1* depletion and its relevance to the E3 ligase activity of WWP2, we further examined the abundance of known WWP2 downstream substrates including PTEN [18] and TRIF [20] upon *Cdh1* depletion, which should inversely correlate with WWP2 enzymatic activity. Notably, we found that in *Cdh1*-depleted cells, the abundance of various WWP2 substrates was dramatically reduced (Figure 1b and c), supporting a model that upon *Cdh1* loss, there is an induction (Supplementary Figure S2b) rather than inactivation (Supplementary Figure S2c) of WWP2 E3 ligase activity. Consistently, depletion of *Cdh1* in DU145 cells resulted in a shortened half-life of WWP2, but not of the other well-characterized Cdh1 substrate, Cdc20 (Supplementary Figure S2d) [24]. These results indicate an elevation of WWP2 activity upon *Cdh1* depletion. In support of this notion, we found that ubiquitination of endogenous WWP2 (Figure 1d) and endogenous PTEN (Figure 1e) were elevated upon *Cdh1* knockdown. More importantly, further depletion of *WWP2* in *Cdh1*-depleted HeLa cells suppressed the elevated ubiquitination of PTEN (Figure 1e). These data altogether suggested that in contrast to its role in promoting Smurf1 activity, Cdh1 may negatively regulate the E3 ligase function of WWP2.

### Cdh1 inhibits the ubiquitin E3 ligase activity of WWP2 in an APC-independent manner

**Figure 2:**
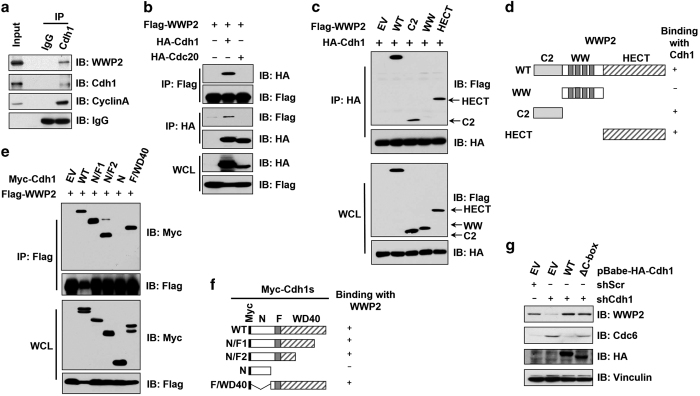
WWP2 interacted with Cdh1 through its C2 and HECT domains. (**a**) Immunoblot (IB) analysis of whole-cell lysates (WCL) and anti-Cdh1 immunoprecipitates (IP) derived from 293 T cells. Mouse IgG was used as a negative control for the IP procedure. (**b**) IB analysis of WCL and IP derived from 293 T cells transfected with HA-Cdh1 or HA-Cdc20 and Flag-WWP2 constructs. Thirty-six hours post transfection, the cells were pretreated with 10 μM MG132 for 10 h before collecting. (**c**) IB analysis of WCL and IP derived from 293 T cells transfected with HA-Cdh1 and the indicated Flag-WWP2 constructs. Thirty-six hours post transfection, the cells were pretreated with 10 μM MG132 for 10 h before collecting. (**d**) A schematic illustration of the WWP2 truncation mutants used in **c**. (**e**) IB analysis of WCL and IP derived from 293 T cells transfected with Flag-WWP2 and the indicated Myc-Cdh1 constructs. Thirty-six hours post transfection, the cells were pretreated with 10 μM MG132 for 10 h before collecting. (**f**) A schematic illustration of the Cdh1 truncation mutants used in **e**. (**g**) IB analysis of MDA-MB-231 cells infected with the indicated lentiviral shRNA constructs together with the indicated pBabe-HA-Cdh1 retroviral constructs. The infected cells were selected with 1 μg ml^−1^ puromycin and 200 μg ml^−1^ hygromycin for 72 h to eliminate the noninfected cells before collecting for IB analysis. shRNA, short hairpin RNA.

In support of this contention, WWP2 interacted with Cdh1 both in cells (Figure 2a) and *in vitro* (Supplementary Figure S3a), but not with another APC co-activator, Cdc20 (Figure 2b). To further pinpoint the binding domains between Cdh1 and WWP2, we generated a series of truncation mutants for both Cdh1 and WWP2. Co-immunoprecipitation experiments indicated that Cdh1 bound to the N-terminal C2 and the C-terminal HECT domains, but not the WW domain of WWP2 (Figure 2c and d). This observation is different from our previous finding that Cdh1 only interacted with the C2 domain, but not the HECT domain of Smurf1 [16]. The different Cdh1 interaction motifs in WWP2 versus Smurf1 might partially explain the opposing role of Cdh1 towards regulating the E3 ligase activity of Smurf1 versus WWP2. On the other hand, we identified the C-terminal WD40 repeats domain of Cdh1 to be responsible for the interaction with WWP2 (Figure 2e and f), through which domain Cdh1 binds to most of its substrates as well as Smurf1 [16].

Given that we previously demonstrated an APC-independent mode of regulating Smurf1 by Cdh1, we further explored the possibility of whether Cdh1 also suppressed WWP2 in an APC-independent manner. To this end, in *Cdh1*-depleted MDA-MB-231 cells (Supplementary Figure S3b), we re-introduced an shCdh1-resistant version of WT-Cdh1 or an E3 ligase activity-deficient ΔC-box-Cdh1, which cannot interact with the APC core complex [4, 25]. Notably, in *Cdh1*-depleted cells, compared with empty vector-infected cells, re-introduction of either WT- or ΔC-box-Cdh1 led to a significant upregulation of WWP2 and subsequent increase of WWP2 substrates including PTEN and TRIF (Figure 2g and Supplementary Figure S3c), arguing that the presence of Cdh1, rather than a functional APC^Cdh1^ E3 ligase is critical in restoring WWP2 abundance. This result indicated that Cdh1 might also govern WWP2 protein levels through an APC-independent manner.

To further elucidate the possible involvement of the APC core complex in controlling WWP2 stability, we depleted various core subunits of the APC complex, such as *APC4*, *APC6* or *APC10*, and found that unlike depletion of *Cdh1*, depleting any of these subunits had minimal effect on WWP2 abundance (Supplementary Figure S3d and i). On the other hand, ectopic expression of WWP2 was unable to trigger Cdh1 degradation (Supplementary Figure S3j), thus excluding Cdh1 as a possible ubiquitin substrate of WWP2. Altogether, these results coherently indicate that similar to Cdh1-mediated regulation of Smurf1, Cdh1 might suppress the E3 ligase activity of WWP2 in an APC-independent manner, possibly through its direct interaction with the C2 and HECT domains of WWP2.

### Cdh1 promotes the intramolecular auto-inhibition of WWP2

**Figure 3:**
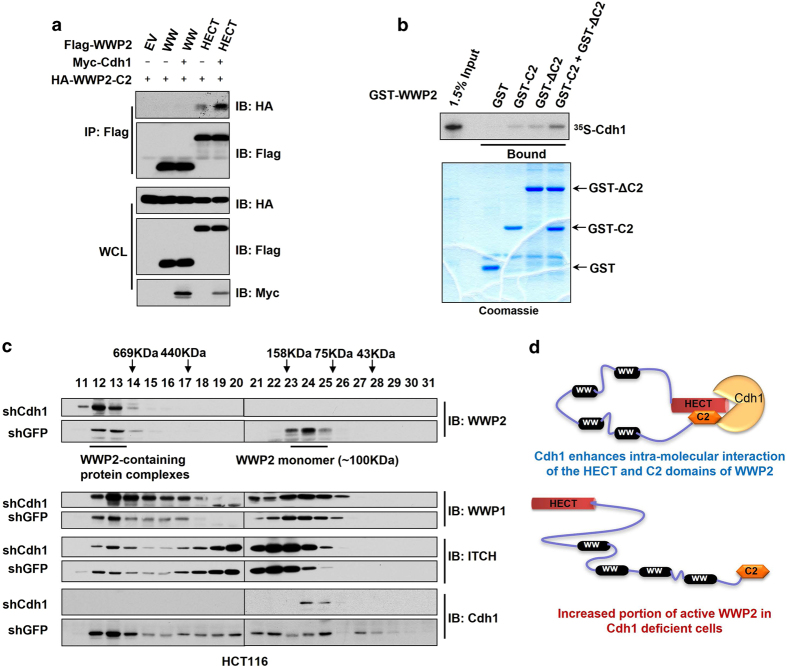
Cdh1 promotes the intramolecular interaction of WWP2. (**a**) Immunoblot (IB) analysis of whole-cell lysates (WCL) and immunoprecipitates (IP) derived from 293 T cells transfected with the indicated Cdh1 and WWP2 constructs. Thirty-six hours post transfection, the cells were pretreated with 10 μM MG132 before collecting. (**b**) Autoradiography of ^35^S-labeled Cdh1 bound to the indicated GST-fusion proteins. (**c**) Gel filtration experiments to illustrate that depletion of endogenous Cdh1 promotes WWP2 oligomer formation in cells. IB analysis of the indicated fractionations derived from the gel filtration experiment with shGFP or shCdh1 infected HCT116 cells. Before running cell lysates, the molecular weight resolution of the column was first estimated by running native molecular weight markers (thyroglobulin ~669 KDa, ferritin ~440 KDa, aldolase ~158 KDa, conalbumin ~75 KDa and ovalbumin ~44 KDa) and determining their retention times on Coomassie-stained SDS–PAGE protein gels. (**d**) A proposed model of how the WWP2 E3 ligase activity is suppressed by Cdh1 via enhancing WWP2 intramolecular interaction-mediated auto-inhibition. PAGE, polyacrylamide gel electrophoresis.

WWP2 has been previously suggested to form an auto-inhibitory conformation through an intramolecular interaction between its C2 and HECT domains [22]. Thus, we sought to further investigate whether Cdh1 could regulate this auto-inhibition to govern the E3 ligase activity of WWP2. Consistent with a previous report [22], we found that the C2 domain could bind the HECT domain but not the WW domain of WWP2 (Figure 3a). Interestingly, the addition of Cdh1 could promote the interaction between the C2 and HECT domains of WWP2 (Figure 3a). In support of this finding, using GST pull-down assays, we found that Cdh1 could bind directly to either the purified C2 domain of WWP2 or the C2 domain-deleted WWP2 (ΔC2), while co-incubation of GST-C2 with GST-ΔC2 promoted their interaction with Cdh1 (Figure 3b).

To further reveal the physiological role of Cdh1 in controlling the monomeric status of WWP2, lysates derived from control or Cdh1-depleted HCT116 cells were fractionated by gel filtration chromatography. Intriguingly, compared with control cells, depletion of endogenous *Cdh1* greatly reduced the monomeric population of WWP2 (fractions 23–25, with estimated molecular weights of ~80–150 KDa), while on the other hand, significantly enhanced the portion of WWP2 within large protein complexes including possible WWP2 dimers or oligomers (fractions 12–13, with estimated molecular weights of ~750 KDa; Figure 3c). Notably, depletion of endogenous *Cdh1* did not significantly alter the migration patterns of other NEDD4 family proteins including WWP1 and ITCH (Figure 3c). Moreover, a dramatic reduction in monomeric population of WWP2, but not WWP1 or ITCH, was similarly observed after depleting *Cdh1* in HeLa cells (Supplementary Figure S4). These data altogether supported a model that Cdh1 binds both the C2 and HECT domains of WWP2 to facilitate the intramolecular interaction, which subsequently inhibits the E3 ligase activity of WWP2 (Figure 3d).

**Figure 4:**
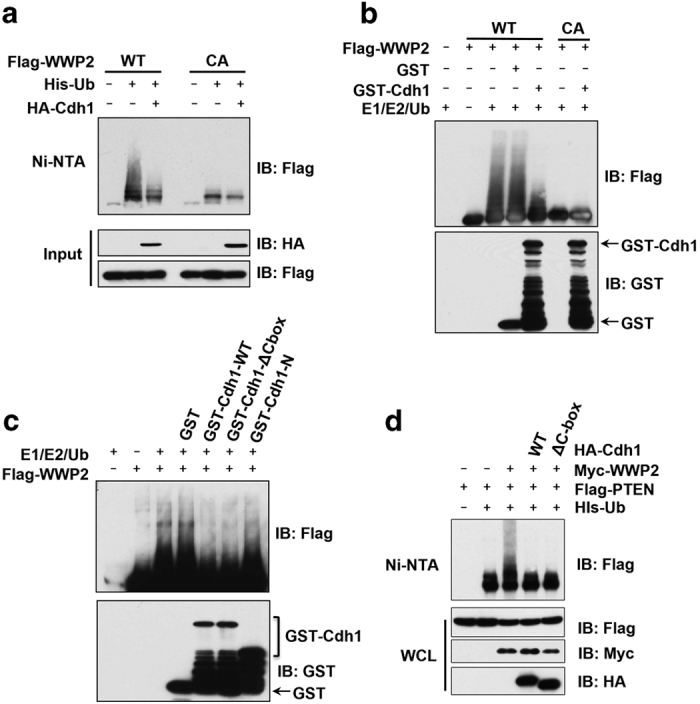
Cdh1 promotes the auto-inhibition of WWP2 E3 ligase. (**a**) Cdh1 suppresses WWP2 auto-ubiquitination in cells. Immunoblot (IB) analysis of whole-cell lysates (WCL) and subsequent Ni-NTA pull-down in 6 M guanidine-HCl-containing buffer derived from 293 T cells transfected with the indicated plasmids. The cells were pretreated with 10 μM MG132 for 10 h before collecting. (**b**) Purified GST-Cdh1 inhibits WWP2 auto-ubiquitination *in vitro*. Bacterially expressed and purified GST-Cdh1 was incubated with E1, E2 and ubiquitin and anti-Flag immunoprecipitates derived from HeLa cells transfected with Flag-WWP2-WT or Flag-WWP2-C838A (CA) constructs as indicated at 30 °C for 15 min. The ubiquitination reaction products were recovered to perform GST pull-down analysis with the indicated GST-fusion proteins then resolved by SDS–PAGE and probed with the indicated antibodies. (**c**) Purified GST-WT-Cdh1 and GST-ΔC-box-Cdh1 inhibits WWP2 auto-ubiquitination *in vitro*. Indicated bacterially purified GST-Cdh1 proteins were incubated with E1, E2 and ubiquitin and anti-Flag immunoprecipitates derived from 293 T cells transfected with Flag-WWP2 constructs as indicated at 30 °C for 15 min. The ubiquitination reaction products were recovered to perform GST pull-down analysis with the indicated GST-fusion proteins then resolved by SDS–PAGE and probed with the indicated antibodies. (**d**) Cdh1 suppresses the E3 ligase activity of WWP2 towards ubiquitinating PTEN in cells. IB analysis of WCL and subsequent Ni-NTA pull-down in 6 M guanidine-HCl-containing buffer derived from 293 T cells transfected with the indicated plasmids. The cells were pretreated with 10 μM MG132 for 10 h before collecting. PAGE, polyacrylamide gel electrophoresis.

To further validate this model, we utilized various biochemical assays to examine the putative role of Cdh1 in regulating the auto-ubiquitination of WWP2. Consistently, we found that auto-ubiquitination of WT-WWP2 could be suppressed by ectopic expression of Cdh1 in cells (Figure 4a). However, C838A-WWP2 (CA), in which the essential cysteine-838 residue was mutated to alanine to abolish its E3 ligase activity [26], could not be ubiquitinated, indicating that the observed ubiquitin ladders were largely via auto-ubiquitination of WWP2 (Figure 4a). Further, *in vitro* ubiquitination assays provided additional support by demonstrating that purified recombinant GST-Cdh1, but not GST, could dramatically reduce the auto-ubiquitination of WWP2 *in vitro* (Figure 4b). More importantly, in further support of an APC-independent regulation of WWP2 by Cdh1 (Figure 1d), both recombinant GST-tagged WT- and ΔC-box-Cdh1 were found to efficiently suppress the auto-ubiquitination of WWP2 *in vitro* (Figure 4c). In contrast, the N-terminal Cdh1, which lacks the WD40 repeats domain [16] that binds WWP2, could not suppress WWP2 auto-ubiquitination (Figure 4c). These results therefore reveal that unlike Cdh1-mediated activation of Smurf1 through interacting specifically with its C2 domain, Cdh1 actively suppresses the E3 ligase activity of WWP2 by directly interacting with both its C2 and HECT domains to enhance the intramolecular C2-HECT interaction.

### Cdh1 suppresses tumorigenesis in part through modulating the WWP2/PTEN/Akt signaling pathway

Next, we sought to determine whether Cdh1 could modulate the protein stability of WWP2 substrates. In keeping with a recent report [18], we found that WT-WWP2, but not the E3 ligase activity-deficient CA-WWP2, could trigger the ubiquitination (Supplementary Figure S5a) and subsequent degradation (Supplementary Figure S5b) of PTEN. Consistently, we demonstrated that depletion of endogenous Cdh1 led to the activation of WWP2 and subsequent downregulation of WWP2 ubiquitin substrates including PTEN and TRIF (Figure 1b and c). To further investigate the critical role of Cdh1 in regulating the WWP2/PTEN/Akt signaling pathway, we continued to assess whether Cdh1 could suppress WWP2-mediated PTEN ubiquitination. Notably, ectopic expression of both WT-Cdh1 and the E3 ligase-deficient Cdh1 mutant, ΔC-box-Cdh1, could dramatically reduce WWP2-mediated PTEN ubiquitination in cells (Figure 4d). These results support the notion that like MC1R [27], Cdh1 might protect PTEN from WWP2-mediated degradation in an APC-independent manner.

**Figure 5:**
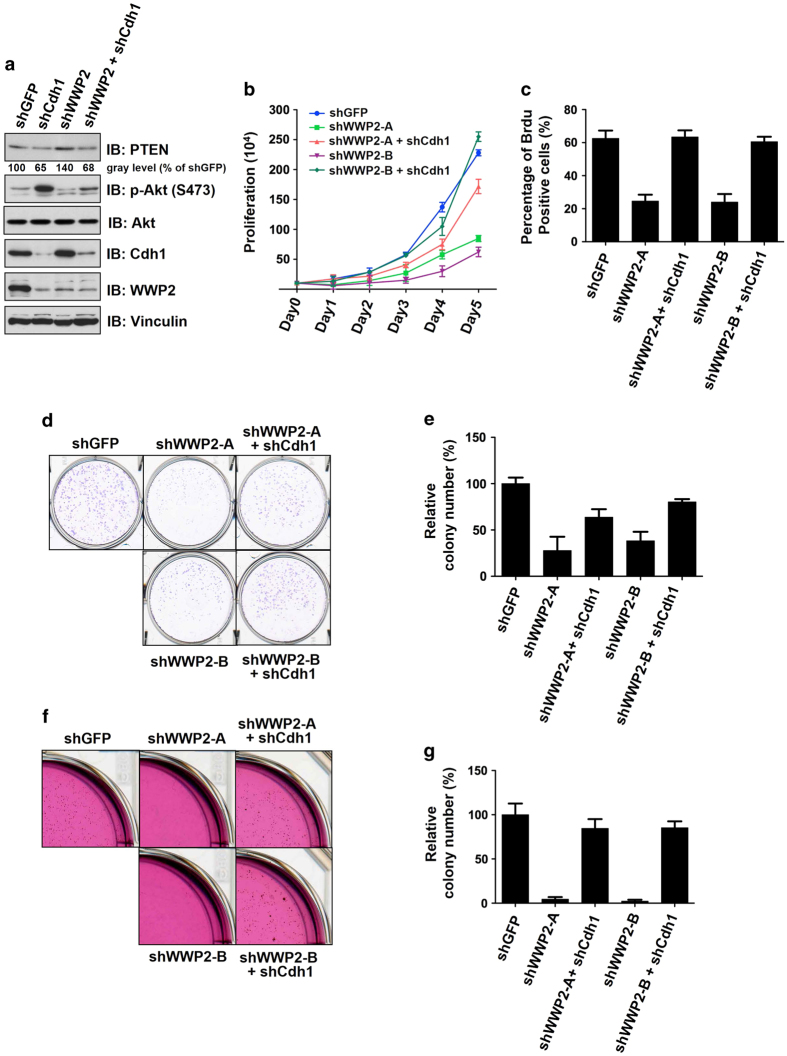
Cdh1 suppresses tumorigenesis partly through modulating the WWP2/PTEN/Akt signaling pathway. (**a**) IB analysis of MDA-MB-231 cells infected with the indicated lentiviral shRNA constructs. The infected cells were selected with 1 μg ml^−1^ puromycin for 72 h to eliminate the noninfected cells before collecting for IB analysis. (**b**) MDA-MB-231 cells stably infected with the indicated lentiviral shRNA constructs were seeded 100 000 cells per well. Afterwards, the cell numbers were counted every day for 5 days to monitor cell proliferation. The data shown are derived from three independent experiments (mean±s.d., *P*<0.05, Student’s *t*-test, compared with cells expressing control shGFP). (**c**) BrdU labeling analysis was performed using MDA-MB-231 cells that were stably infected with the indicated lentiviral shRNA constructs. The cells were seeded 50 000 cells per well; 24 h later, the cells were incubated with BrdU and uridine for 48 h before photographs were taken. Percentage of BrdU positive cells was illustrated as mean±s.e.m. (*n*=3). (**d**, **e**). MDA-MB-231 cells stably infected with the indicated lentiviral shRNA constructs were seeded 10 000 cells per well. Seven days later, crystal violet was used to stain the formed colonies before photographs were taken (**d**). Colony numbers were counted from three independent experiments and the relative colony numbers were calculated as mean±s.e.m. (*n*=3) in **e**. (**f**, **g**) MDA-MB-231 cells stably infected with the indicated lentiviral shRNA constructs were seeded (3 000 cells per well) in 0.5% low-melting-point agarose in DMEM with 10% FBS and layered onto 0.8% agarose in DMEM+10% FBS. The plates were cultured for 30 days before photographs were taken (**f**). The colonies >50 μm were counted under a light microscope. The relative colony numbers were plotted as mean±s.e.m. from three independent experiments (**g**). BrDU, bromodeoxyuridine; DMEM, Dulbecco’s Modified Eagle’s medium; IB, immunoblotting; FBS, fetal bovine serum; shRNA, short hairpin RNA.

In further support of this hypothesis, we found that depleting endogenous *Cdh1* in MDA-MB-231 breast cancer cells led to downregulation of PTEN and subsequent elevation of Akt activity as demonstrated by an increase in pS473-Akt (Figure 5a). More importantly, further depletion of *WWP2* partly rescued Cdh1 depletion-induced PTEN suppression and Akt activation (Figure 5a). These results therefore indicate that the increase of pS473-Akt levels after *Cdh1* knockdown might be at least in part through activation of the WWP2 E3 ligase activity, which in turn led to a decrease in PTEN abundance. Importantly, aberrant activation of the PI3K/Akt signaling pathway has been observed in many types of human cancers [28], therefore a possible involvement of Cdh1 in negatively regulating this critical oncogenic pathway may underscore the emerging tumor suppressive role of Cdh1 [24].

We next sought to investigate how the Cdh1/WWP2 signaling axis impinges on the tumorigenicity of cancer cells. In concert with a previous report [18], depletion of *WWP2* significantly suppressed pS473-Akt levels in MDA-MB-231 cells (Supplementary Figure S5c). As a consequence, *WWP2*-depleted cells displayed a dramatic decrease in proliferation as evidenced by both cell growth assays (Figure 5b) and bromodeoxyuridine (BrdU) incorporation assays (Figure 5c and Supplementary Figure S5d). Notably, further depletion of *Cdh1* could partly restore the decrease in both cell growth (Figure 5b) and BrdU incorporation (Figure 5c and Supplementary Figure S5d) in *WWP2*-depleted cells, suggesting a crucial physiological role of Cdh1 in regulating WWP2-mediated PTEN downregulation and subsequent Akt activation to influence cell growth.

**Figure 6:**
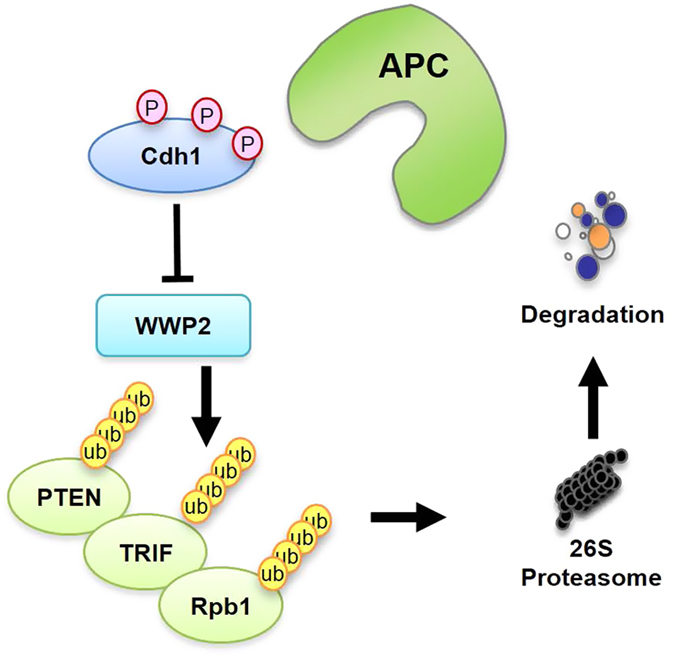
A schematic illustration of the proposed model of how Cdh1 exerts its tumor suppressor role in part by the negative regulation of WWP2 E3 ligase activity to stabilize PTEN and to suppress the oncogenic Akt signaling. In most cancer cells, where Cdh1 is largely APC-free due to relatively high Cdk activity, Cdh1 mainly directly binds and inhibits WWP2, which leads to the accumulation of various WWP2 substrates including PTEN, TRIF and Rpb1. But further phosphorylation of Cdh1 by Cdk and Plk promotes Cdh1 degradation and the loss of Cdh1 eventually leads to PTEN destabilization and subsequent Akt activation to facilitate tumorigenesis. APC, anaphase-promoting complex/cyclosome.

Consistently, *WWP2*-depleted cells formed less colonies compared with control cells, while further depletion of *Cdh1* largely restored the growth potential of these cells in both colony formation (Figure 5d and e) and soft agar assays (Figure 5f and g). These results demonstrated that Cdh1 might inhibit tumorigenesis partly through inhibiting WWP2 E3 ligase activity, which led to PTEN accumulation and subsequent PI3K/Akt suppression. As elevated Cdk2/cyclin A and Cdk2/cyclin E activities, which are tightly associated with tumorigenesis, has been reported to dissociate Cdh1 from the APC core complex [29], it is possible that in tumor cell settings, a large portion of Cdh1 exists in an APC-free mode to modulate tumorigenesis by governing the WWP2/PTEN/Akt signaling axis (Figure 6 and Supplementary Figure S6). The different distribution of APC-free and APC-bound Cdh1 in primary somatic cells versus tumor cells (Supplementary Figure S6) further indicates that Cdh1 might function differently via an APC-dependent or APC-independent mechanism in different cellular contexts, such as somatic versus tumor cells (Figure 6).

## Discussion

The central roles of APC^Cdh1^ in cell cycle regulation, primarily in controlling the G1/S transition and DNA replication, have been well documented [30–33]. In addition to its role in cell cycle control, APC^Cdh1^ has been implicated in regulating genomic integrity, cellular differentiation and pathogenesis of various diseases [34, 35]. These functions of APC^Cdh1^ are implemented predominantly through timely ubiquitination and subsequent degradation of its substrates and, therefore, rely on the integrity of the APC holoenzyme [36]. However, Cdh1 only associates with the APC core complex from early-to-mid G1 phase; during the rest of cell cycle, Cdh1 is largely APC-free in part owing to Cdk-mediated phosphorylation of Cdh1 [37]. This prompted us to investigate the possible roles of Cdh1 independent of the APC core complex. To this end, our previous study demonstrated that Cdh1 augmented Smurf1 E3 ligase activity in an APC-independent manner, to govern osteoblast differentiation [16] (Supplementary Figure S2a). In this present study, we further found that Cdh1 could also regulate the E3 ligase activity of another NEDD4 family member WWP2, independent of the APC E3 ligase activity (Supplementary Figure S2b).

Interestingly, in contrast to its positive regulation on Smurf1, we found that Cdh1 inhibited WWP2 (Supplementary Figure S2b). The opposite roles of Cdh1 in controlling Smurf1 and WWP2 activity might be explained by the distinct inhibitory mechanisms that Smurf1 and WWP2 adopt. Similar to Smurf2 and ITCH, WWP2 tends to form an intramolecular inhibitory conformation with the N-terminal C2 domain binding to the C-terminal HECT domain within the same molecule [22]. However, it might be unfavorable for Smurf1, with only two WW motifs between the C2 and HECT domains, to form the intramolecular interaction found in other NEDD4 family members, most of which have four WW motifs to fully separate the C2 and HECT domains. Instead, we previously showed that Smurf1 formed a head-to-toe intermolecular dimer, rather than an intramolecular conformation, which also inhibited its E3 ligase activity [16]. Cdh1, on the other hand, binds the C2 domain of Smurf1, and thereby competing with the HECT domain from the other Smurf1 molecule. In this regard, Cdh1 could break the Smurf1 inhibitory dimer to activate Smurf1. Importantly, here we demonstrated that Cdh1 interacted with WWP2 via both its C2 and HECT domains, which indicated a model that Cdh1 might facilitate WWP2 intramolecular inhibition to negatively regulate its E3 ligase activity (Figure 3d). This similar, albeit functionally opposite regulation mechanism, illustrated a complicated role for Cdh1 in controlling the NEDD4 family proteins. However, further studies are warranted to further assess a possible role for Cdh1 in modulating the activity of other NEDD4 family members, or C2 domain-containing E3 ligases.

As an ubiquitin E3 ligase, WWP2 exerts its physiological functions primarily through tagging its substrates with ubiquitin to regulate downstream signaling pathways. Hence, in different cellular or tissue contexts with distinct proteomic profiles, WWP2 may function differently. This context-dependent physiological function of E3 ligases has been shown previously for other E3 ligases, including SCF^β-TRCP^ [38] and APC^Cdh1^ [39, 40]. Furthermore, Cdh1 has been identified as a Cdk substrate [41–44], and the phosphorylation of Cdh1 by Cdk2-cyclin A/E in late G1/S phases and by Cdk1-cyclin B in G2/M phases abolishes the interaction of Cdh1 with the APC core complex [29]. As elevation of Cdk activity has been found in most cancer cells [45], Cdh1 might largely exist in an APC-free population in these cancer cells, which directly interacts with WWP2 to facilitate its auto-inhibition (Figure 3d).

In support of this notion, we found that in ATDC5 cells, Cdh1 existed mainly in fractions with molecular weight over 660 KDa, with little protein migrated in the monomeric fractions (Supplementary Figure S6), suggesting Cdh1 was predominantly associated with APC in ATDC5 cells. More importantly, the co-migration of Cdh1 and WWP2, which should appear in the fractions of molecular weight around 150 KDa, was undetectable in ATDC5 cells (Supplementary Figure S6). On the other hand, in various cancer cells including HCT116 (Figure 3c), HeLa (Supplementary Figure S4) and MDA-MB-231 (Supplementary Figure S6), Cdh1 migrated in most of the fractions ranged from 50 KDa to over 200 KDa, and overlapped with WWP2 around 150 KDa, indicating that in cancer cells, a significant portion of Cdh1 is APC-free, which might bind WWP2 to inhibit the E3 ligase activity of WWP2 (Figure 6).

The suppression of WWP2 activity by APC-free Cdh1 led to an accumulation of PTEN and thereby attenuated the oncogenic PI3K/Akt signaling cascade. Furthermore, during tumor progression, owing to attenuated APC^Cdh1^ activity by Cdk phosphorylation, various APC^Cdh1^ substrates including cyclins and mitotic kinases are accumulated, which not only leads to further suppression of APC^Cdh1^ activity, but possibly also triggers Cdh1 destruction by SCF^β-TRCP^ [41] to release WWP2 E3 ligase activity for PTEN degradation and subsequent PI3K/Akt activation. Hence, our finding provided further evidence to demonstrate the putative role for Cdh1 as a tumor suppressor [24] and indicated that reduced expression of Cdh1 in certain types of human cancers [13] might be a biomarker to predict the elevation of PI3K/Akt activities.

Taken together, our results provided a mechanistic insight into a novel molecular mechanism that Cdh1 suppresses tumorigenesis by augmenting the intramolecular interaction between the HECT and C2 domains of WWP2 to lock the WWP2 E3 ligase in an inactive form (Figure 3d). Interestingly, we demonstrated that this regulation is independent of the E3 ligase activity of Cdh1. Therefore, inducing Cdh1 abundance may present a potential therapeutic approach for the treatment of human cancers with high E3 ligase activity of WWP2.

## Materials and Methods

### Plasmids

pLKO-shRNA (short hairpin RNA) constructs against human WWP2 (RHS4533-EG11060), human APC4 (RHS4533-EG29945), human APC6 (RMM4534-EG69957) and human APC10 (RHS4533-EG10393) were purchased from Open Biosystems (Lafayette, CO, USA). pFlag-WWP1, pFlag-WWP2 and pFlag-ITCH were made by inserting digested PCR products into pFlag-CMV2 vector. pCI-HA-NEDD4 (27002) and pCI-HA-NEDD4L (27000) [46] were purchased from Addgene (Cambridge, MA, USA) and were subcloned into pCMV-Flag to generate Flag-NEDD4 and Flag-NEDD4L, respectively. His-Ubiquitin, HA-Cdh1, HA-Cdc20, Myc-Cdh1, pBabe-HA-Cdh1, pGEX-Cdh1, Flag-Smurf1 and Flag-Smurf2 were described previously [16]. To generate pGEX-4T-1-WWP2, human WWP2 cDNA was subcloned into pGEX-4T-1 vector. The targeting sequences of pLKO lentiviral constructs to deplete human Cdh1 are: shCdh1-A (sense: 5′-GGCAACGATGTGTCTCCCT-3′), shCdh1-B (sense: 5′-CAGCCTTGTTTCTCATGTA-3′).

### Antibodies

Anti-ITCH antibody (611198) was purchased from BD Labs (Waltham, MA, USA). Anti-WWP1 antibody (A302-949A), anti-WWP2 antibody (A302-935A), anti-APC4 antibody (A301-176A), anti-APC6 antibody (A301-165A) were purchased from Bethyl Labs (Montgomery, TX, USA). Anti-Aurora A antibody (3092), anti-PTEN antibody (9188) anti-NEDD4L antibody (4013), anti-NEDD4 antibody (2740), anti-TRIF antibody (4596), anti-phospho-Ser473-Akt antibody (4051) and anti-pS9-GSK3β antibody (9323) were purchased from Cell Signaling (Danvers, MA, USA). Anti-Smurf1 antibody (45-K) (H-60), anti-cyclin A antibody (H-432), anti-Plk1 antibody (F-8), anti-APC10 antibody (B-1), anti-c-Myc antibody (9E10) and polyclonal anti-HA antibody (Y-11) were purchased from Santa Cruz. Anti-tubulin antibody (T-5168), anti-vinculin antibody (V-4505), polyclonal anti-Flag antibody (F-2425), monoclonal anti-Flag antibody (F-3165), anti-Flag agarose beads (A-2220), anti-HA agarose beads (A-2095), peroxidase-conjugated anti-mouse secondary antibody (A-4416) and peroxidase-conjugated anti-rabbit secondary antibody (A-4914) were purchased from Sigma (St Louis, MO, USA). Monoclonal anti-HA antibody (MMS-101P) was purchased from Covance (Dedham, MA, USA). Anti-GFP antibody (632380) and polyclonal anti-Cdh1 antibody (34–2000) were purchased from Invitrogen (Carlsbad, CA, USA). Monoclonal anti-Cdh1 (CC43) was purchased from Calbiochem (San Diego, CA, USA).

### Cell culture, transfection and infection

Cell culture conditions for HeLa, 293 T, MDA-MB-231, DU145, PC3, T98G and U2OS cells have been described previously [16]. Briefly, cells are cultured in Dulbecco’s Modified Eagle’s medium (DMEM) media containing 10% fetal bovine serum (FBS) and Pen/Strep in 5% CO_2_ cell culture incubator. ATDC5 cell culture condition was described previously[47] that ATDC5 cells are cultured in DMEM:Ham’s F12 (1:1) media supplemented with 2 mM glutamine, 5% FBS and Pen/Strep in 5% CO_2_ cell culture incubator. Cell transfection using lipofectamine reagent (Thermo Fisher Scientific, Waltham, MA, USA) is performed according to manufacturer’s instructions.

Lentiviral shRNA virus packaging and subsequent infection of various cell lines were performed according to the protocol described previously [48]. Lentiviral shRNA virus are packaged by transfecting 293 T cells with pLKO.1-shRNA constructs together with pCMV-dR8.91 (Delta 8.9) plasmid containing gag, pol and rev genes and VSV-G expressing envelope plasmid. Virus-containing media were harvested 48 h post transfection and filtered before being used for infection. Lentiviral infection was performed using virus-containing media with 4 μg ml^−1^ polybrene for 24 h before selection by puromycin.

### Immunoblots and immunoprecipitation

Cells were lysed in EBC buffer (50 mM Tris pH 7.5, 120 mM NaCl, 0.5% NP-40) supplemented with protease inhibitors (Complete Mini, Roche, Basel, Switzerland) and phosphatase inhibitors (phosphatase inhibitor cocktail set I and II, Calbiochem). The protein concentrations of the lysates were measured using the Bio-Rad protein assay reagent (Bio-Rad, Waltham, MA, USA) on a Beckman Coulter DU-800 spectrophotometer (Beckman Coulter, Danvers, MA, USA). The lysates were then resolved by SDS–polyacrylamide gel electrophoresis (SDS–PAGE) and immunoblotted with indicated antibodies. For immunoprecipitation, 800 μg lysates were incubated with the appropriate antibody (1–2 μg) for 3–4 h at 4 °C followed by 1-h incubation with Protein A sepharose beads (GE Healthcare, Little Chalfont, UK). Immuno-complexes were washed five times with NETN buffer (20 mM Tris, pH 8.0, 100 mM NaCl, 1 mM EDTA and 0.5% NP-40) before being resolved by SDS–PAGE and immunoblotted with indicated antibodies.

### *In vitro* binding assays

Binding to immobilized GST proteins was performed as described previously [8, 49]. Briefly, GST-WWP2 proteins were expressed in the BL21 *E.coli* strain followed by purification using Glutathione Sepharose 4B (GE Healthcare #17-0756-01) according to manufacturer’s instructions. Purified GST and GST-WWP2 proteins were incubated with ^35^S-labeled *in vitro* transcribed and translated Cdh1 in NETN buffer (20 mM Tris, pH 8.0, 100 mM NaCl, 1 mM EDTA and 0.5% NP-40) for 3–4 h at 4 °C followed by washing with NETN buffer before being resolved by SDS–PAGE and immunoblotted with indicated antibodies.

### Gel filtration chromatography analysis

HeLa and HCT116 cells were infected with shCdh1 and shGFP lentiviral constructs. Seventy-two hours post infection, the cells were washed with phosphate-buffered saline (PBS), lysed in 0.5 ml of EBC buffer (50 mM Tris pH 7.5, 120 mM NaCl, 0.5% NP-40) containing protease inhibitors (Complete Mini, Roche) and phosphatase inhibitors (phosphatase inhibitor cocktail set I and II, Calbiochem), and filtered through a 0.45 μm syringe filter. Total protein concentration was then adjusted to 8 mg ml^−1^ with EBC buffer and 500 μl of the lysate was loaded onto a Superdex 200 10/300 GL column (GE Life Sciences Cat. No. 17-5175-01, Pittsburgh, PA, USA). Chromatography was performed on the AKTA-FPLC (GE Life Sciences Cat. No. 18-1900-26) with EBC buffer as described previously [16]. One column volume of elutes was fractionated with 500 ml in each fraction, at the elution speed of 0.5 ml min^−1^. Thirty-microliter aliquots of each fraction were loaded onto SDS–PAGE gels and detected with indicated antibodies.

### Ubiquitination analysis in cells

WWP2 and APC^Cdh1^ ubiquitination assays in cells were performed as described previously [16, 47]. Briefly, for WWP2 ubiquitination assays in cells, 293 T cells were transfected with Flag-WWP2, His-ubiquitin and HA-Cdh1. Thirty-six hours after transfection, 10 μM MG132 was added to block proteasome degradation, and cells were collected in EBC buffer containing protease inhibitors. Whole-cell lysates (2 mg) were incubated with Ni-NTA beads for 4 h, followed by washing four times with NETN buffer. Then the washed pellet was boiled in SDS-containing sample buffer and resolved by SDS–PAGE.

### *In vitro* ubiquitination analysis

WWP2 *in vitro* ubiquitination assays were performed with protocols described previously [16]. Briefly, Flag-WT-WWP2 and Flag-CA-WWP2 proteins were expressed and immuno-purified in 293 T cells. GST-WT-Cdh1, GST-ΔC-box-Cdh1 and GST-N174-Cdh1 proteins were expressed and purified in BL21 (DE3) *E. coli*. The WWP2 *in vitro* auto-ubiquitination assay was performed in 20 μl ubiquitination assay buffer (50 mM Tris-HCl pH 8.0, 50 mM NaCl, 1.5 mM dithiothreitol, 50 mM MgCl_2_, 10 mM ATP), with 0.5 μg of E1, 1 μg of UbcH5C (E2), 1.5 μg of HA-ubiquitin (Boston Biochem, Cambridge, MA, USA). A total 150 ng Flag-WWP2 (wild type or mutant) together with 500 ng of GST, GST-WT-Cdh1, GST-ΔC-box-Cdh1 or GST-N174-Cdh1 was added to the buffer to initiate the reaction. The samples were incubated at 30 °C for 15 min. The reactions were stopped by the addition of 2× SDS–PAGE sample buffer, and the reaction products were resolved by SDS–PAGE and probed with indicated antibodies.

### Clonogenic survival and soft agar assays

For clonogenic survival assays, 1 000 cells were plated into six-well plates with the indicated chemical treatment. After 14 days, the cells were stained using crystal violet and colonies larger than 50 cells were stained and quantified as described previously [27].

For soft agar assays, cells (3 000 per well) were seeded in 0.5% low-melting-point agarose in DMEM with 10% FBS, layered onto 0.8% agarose in DMEM/10% FBS. The plates were kept in the cell culture incubator for 30 days after which the colonies >50 μm were counted under a light microscope.

### Bromodeoxyuridine labeling

MDA-MB-231 cells were incubated with BrdU and uridine for 48 h. Then BrdU labeling assay was performed as described before [50]. Briefly, cultures were incubated with 1 mg ml^−1^ (3 mM) BrdU and 1 mg ml^−1^ uridine for 48 h. The cells were washed with PBS, fixed with ice-cold absolute methanol for 10 min, treated with 1.5 M HCl for 1 h at room temperature, and neutralized with 0.1 M borate buffer (pH 8.5) for 30 min. After blocking with 0.1% bovine serum albumin in PBS for 30 min at 37 °C, the cells were incubated with 5 mg ml^−1^ anti-BrdU monoclonal antibody (Pharmingen, Franklin Lakes, NJ, USA) in 0.1% PBS/bovine serum albumin for 1 h, washed with PBS/bovine serum albumin and incubated with 1 mg ml^−1^ HRP-conjugated rabbit anti-mouse secondary antibody (Jackson ImmunoResearch, West Grove, PA, USA) for 1 h. The cells were then washed extensively with ammonia-buffered phosphate (0.1 M NaH_2_PO_4_ brought to pH 7.0 with ammonium hydroxide) and stained for 12–16 h at room temperature with 1.3 mM 3,39-diaminobenzidine in ammonia-buffered phosphate containing 0.004% H_2_O_2_.

### Statistical analysis

All quantitative data were presented as the mean±s.e.m. or the mean±s.d. as indicated of at least three independent experiments by Student’s *t*-test for between-group differences. The *P*<0.05 was considered as statistically significant.
