# Supplementary material for: Cdh1 inhibits WWP2-mediated ubiquitination of PTEN to suppress tumorigenesis in an APC-independent manner
Source: Cell Discov. 2016 Feb 2;2:15044–. doi: 10.1038/celldisc.2015.44 (PMC4860961; doi:10.1038/celldisc.2015.44)
Supplement: Supplementary Figure S1 [file celldisc201544-s1.pdf]

Supplementary Figure 1

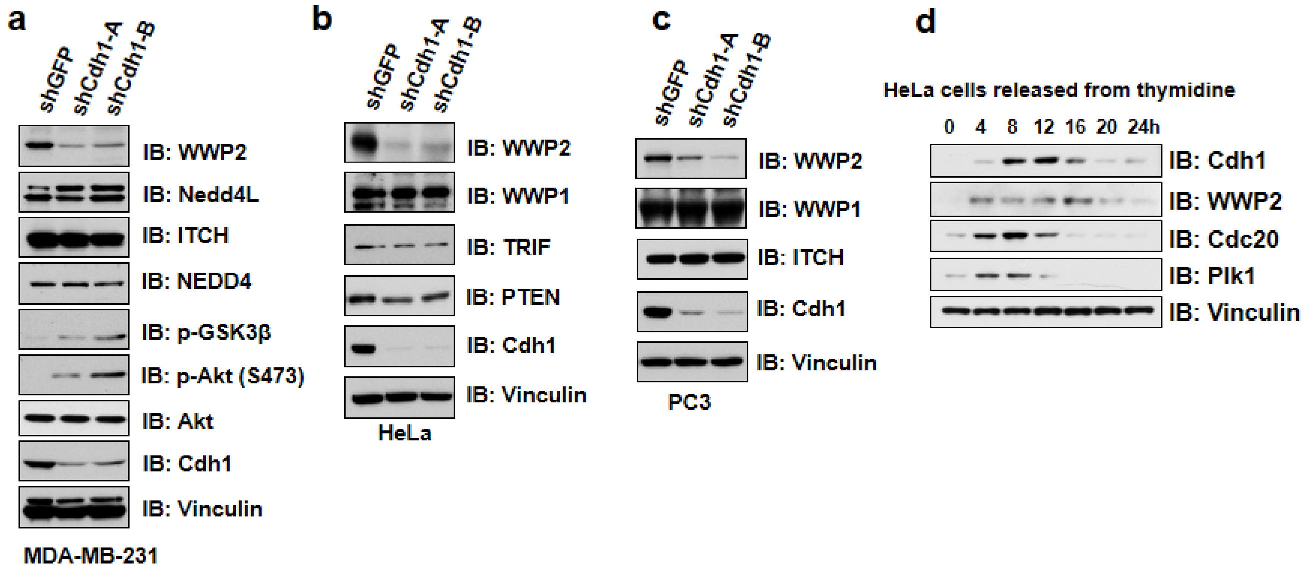

Supplementary Figure 1. Depletion of endogenous *Cdh1* suppresses the protein levels of WWP2.

a. Immunoblot (IB) analysis of MDA-MB-231 cells infected with the indicated lentiviral shRNA constructs. The infected cells were selected with 1 μg/ml puromycin for 72 hours to eliminate the non-infected cells before harvesting for IB analysis.

b. IB analysis of HeLa cells infected with the indicated lentiviral shRNA constructs. The infected cells were selected with 1 μg/ml puromycin for 72 hours to eliminate the non-infected cells before harvesting for IB analysis.

c. IB analysis of PC3 cells infected with the indicated lentiviral shRNA constructs. The infected cells were selected with 1 μg/ml puromycin for 72 hours to eliminate the non-infected cells before harvesting for IB analysis.

d. IB analysis of WCL derived from HeLa cells synchronized at the G1/S boundary by double-thymidine block then released back into the cell cycle for the indicated periods of time.
