# Supplementary material for: Cdh1 inhibits WWP2-mediated ubiquitination of PTEN to suppress tumorigenesis in an APC-independent manner
Source: Cell Discov. 2016 Feb 2;2:15044–. doi: 10.1038/celldisc.2015.44 (PMC4860961; doi:10.1038/celldisc.2015.44)
Supplement: Supplementary Figure S2 [file celldisc201544-s2.pdf]

Supplementary Figure 2

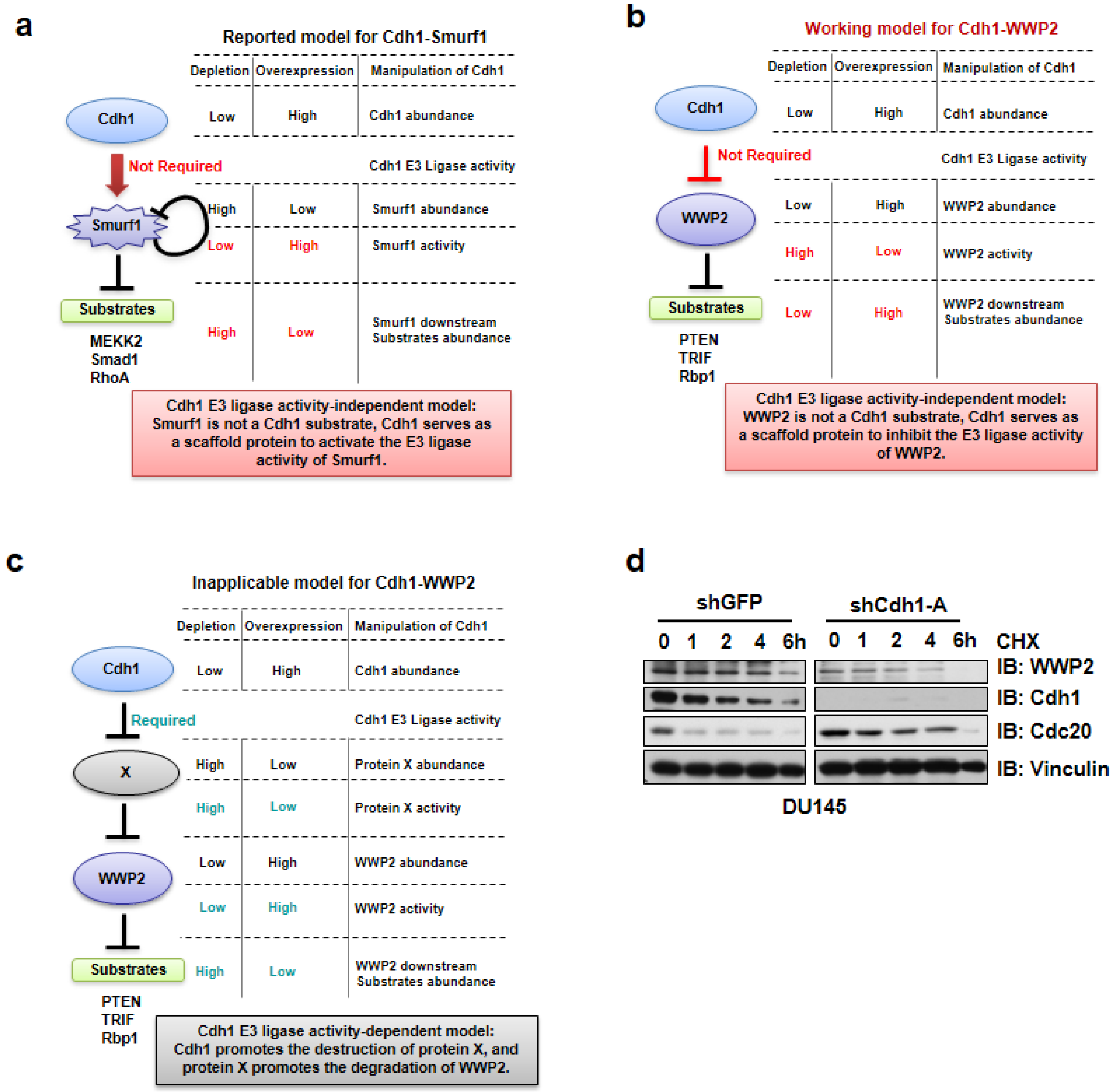

Supplementary Figure 2. Schematic illustrations of models for how Cdh1 modulates the E3 ligase activities of Smurf1 and WWP2 via different possible mechanisms.

a. A schematic illustration of the models for how Cdh1 augments Smurf1 E3 ligase activity and its downstream signaling pathway.

b-c. Schematic illustrations of two possible models for how Cdh1 modulates WWP2 E3 ligase activity and its downstream signaling pathway.

d. Immunoblot (IB) analysis of whole cell lysates (WCL) derived from DU145 cells infected with the indicated lentiviral shRNA constructs for 24 hours. Non-infected cells were eliminated by selection with 1 µg/ml puromycin for 48 hours. 20 µg/ml cycloheximide (CHX) was added to the resulting cells for the indicated time periods before harvesting for IB analysis.
