# Supplementary material for: Cdh1 inhibits WWP2-mediated ubiquitination of PTEN to suppress tumorigenesis in an APC-independent manner
Source: Cell Discov. 2016 Feb 2;2:15044–. doi: 10.1038/celldisc.2015.44 (PMC4860961; doi:10.1038/celldisc.2015.44)
Supplement: Supplementary Figure S3 [file celldisc201544-s3.pdf]

Supplementary Figure 3

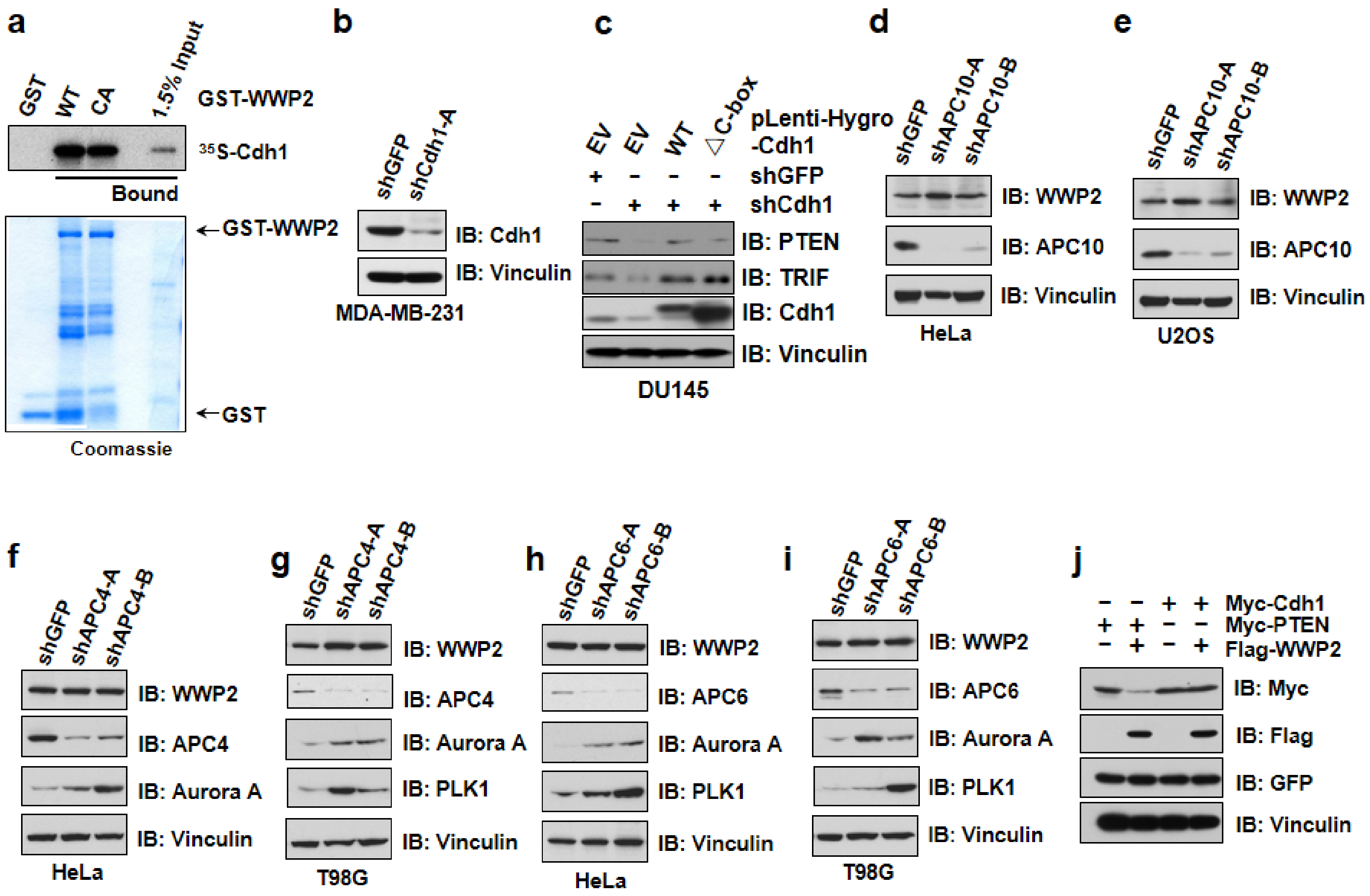

Supplementary Figure 3. Cdh1 inhibits the E3 ligase activity of WWP2 in an APC-independent manner.

- **a.** Autoradiography of ^{35}S-labelled Cdh1 bound to the indicated GST fusion proteins.
- **b.** Immunoblot (IB) analysis of *Cdh1* depletion in shGFP and shCdh1-MDA-MB-231 cells.
- **c.** IB analysis of DU145 cells infected with the indicated lentiviral constructs. The infected cells were selected with 1 µg/ml puromycin and 200 µg/ml hygromycin for 72 hours to eliminate the non-infected cells before harvesting for IB analysis.
- **d-e.** IB analysis of HeLa (**d**) or U2OS (**e**) cells infected with the indicated lentiviral shAPC10 constructs (with shGFP as a negative control). The infected cells were selected with 1 µg/ml puromycin for 72 hours to eliminate the non-infected cells before harvesting for IB analysis.
- **f-g**. IB analysis of HeLa (**f**) or T98G (**g**) cells infected with the indicated lentiviral shAPC4 constructs (with shGFP as a negative control). The infected cells were selected with 1 µg/ml puromycin for 72 hours to eliminate the non-infected cells before harvesting for IB analysis.
- **h-i.** IB analysis of HeLa (**h**) or T98G (**i**) cells infected with the lentiviral shAPC6 constructs (with shGFP as a negative control). The infected cells were selected with 1 µg/ml puromycin for 72 hours to eliminate the non-infected cells before harvesting for IB analysis.
- **j.** IB analysis of WCL derived from 293T cells transfected with Myc-Cdh1 or Myc-PTEN with Flag-WWP2 constructs.
