# Supplementary material for: Cdh1 inhibits WWP2-mediated ubiquitination of PTEN to suppress tumorigenesis in an APC-independent manner
Source: Cell Discov. 2016 Feb 2;2:15044–. doi: 10.1038/celldisc.2015.44 (PMC4860961; doi:10.1038/celldisc.2015.44)
Supplement: Supplementary Figure S4 [file celldisc201544-s4.pdf]

Supplementary Figure 4

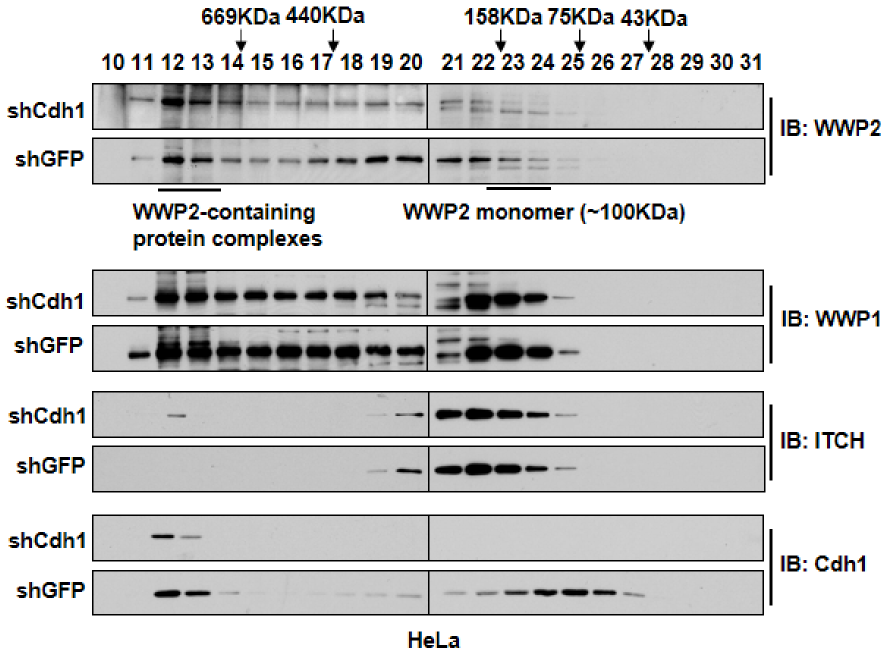

Supplementary Figure 4: Gel filtration experiment to illustrate that Cdh1 depletion leads to decreased WWP2 monomeric fractions. Immunoblot (IB) analysis of the indicated fractionations derived from the gel filtration experiment with shGFP- or shCdh1-HeLa cells, and whole cell lysates were harvested using EBC buffer. Prior to running cell lysates, the molecular weight resolution of the column was first estimated by running native molecular weight markers (Thyroglobulin ~669KD, Ferritin ~440KD, Aldolase ~158KD, Conalbumin ~75KD and Ovalbumin ~44KD) and determining their retention times on coomassie-stained SDS-PAGE protein gels.
