# Supplementary material for: Cdh1 inhibits WWP2-mediated ubiquitination of PTEN to suppress tumorigenesis in an APC-independent manner
Source: Cell Discov. 2016 Feb 2;2:15044–. doi: 10.1038/celldisc.2015.44 (PMC4860961; doi:10.1038/celldisc.2015.44)
Supplement: Supplementary Figure S5 [file celldisc201544-s5.pdf]

Supplementary Figure 5

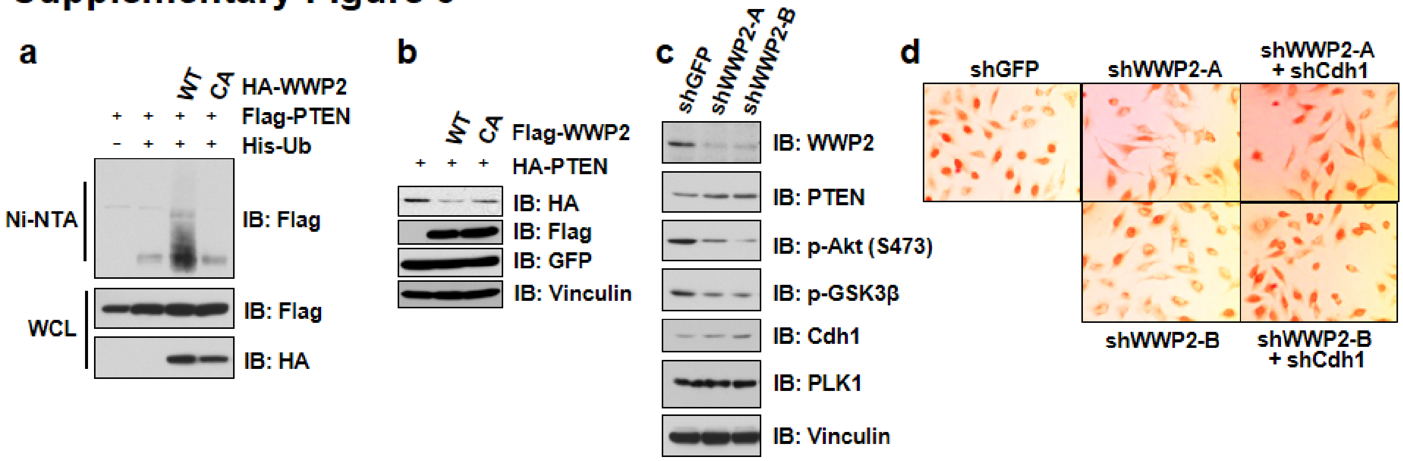

Supplementary Figure 5. Cdh1 suppresses tumorigenesis partly through modulating the WWP2/PTEN/Akt signaling pathway.

a. WWP2 targets PTEN for ubiquitination in cells. Immunoblot (IB) analysis of whole cell lysates (WCL) and subsequent Ni-NTA pull-down in 6 M guanidine-HCl containing buffer derived from 293T cells transfected with the indicated plasmids. Cells were pre-treated with 10 μM MG132 for 10 hours before harvesting.

b. IB analysis of WCL derived from 293T cells transfected with Flag-WWP2-WT or Flag-WWP2-CA together with HA-PTEN constructs.

c. IB analysis of MDA-MB-231 cells infected with the indicated lentiviral shRNA constructs. The infected cells were selected with 1 μg/ml puromycin for 72 hours to eliminate the non-infected cells before harvesting for IB analysis.

d. BrdU labeling analysis was performed using MDA-MB-231 cells that were stably infected with the indicated lentiviral shRNA constructs. Cells were seeded 50,000 cells per well, 24 hours later, the cells were incubated with BrdU and uridine for 48 hours before taken photographs.
