# Supplementary material for: Cdh1 inhibits WWP2-mediated ubiquitination of PTEN to suppress tumorigenesis in an APC-independent manner
Source: Cell Discov. 2016 Feb 2;2:15044–. doi: 10.1038/celldisc.2015.44 (PMC4860961; doi:10.1038/celldisc.2015.44)
Supplement: Supplementary Figure S6 [file celldisc201544-s6.pdf]

Supplementary Figure 6

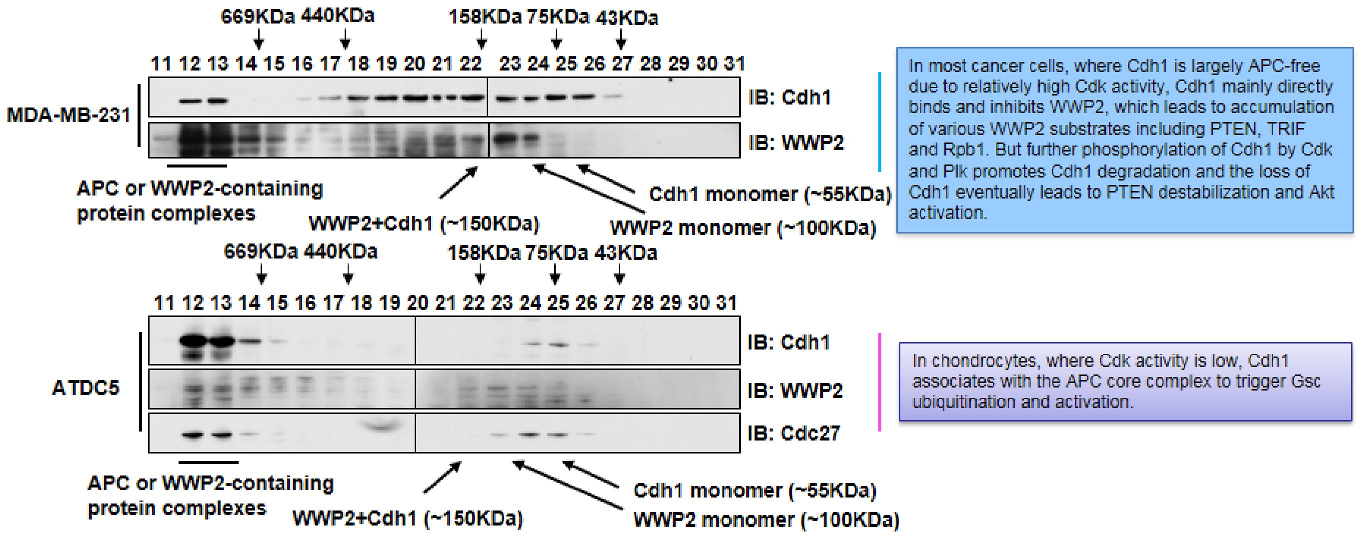

Supplementary Figure 6. Gel filtration experiment to illustrate the different distribution of APC-free and APC-bound Cdh1 in different cellular contexts. IB analysis of the indicated fractionations derived from gel filtration experiments using MDA-MB-231 (top panel, a cancer cell line) or ATDC5 (lower panel, a primary chondrocyte cell line) cells to demonstrate that different from the obvious co-migration of Cdh1 and WWP in cancer cells, there was no detectable co-migration fractions for Cdh1 and WWP2 in ATDC5 cells.
